# Impact of Multiple Chronic Conditions on Activity Limitations Among Older Mexican-American Care Recipients

**DOI:** 10.5888/pcd15.170358

**Published:** 2018-05-03

**Authors:** Diane M. Collins, Brian Downer, Amit Kumar, Shilpa Krishnan, Chih-ying Li, Kyriakos S. Markides, Amol M. Karmarkar

**Affiliations:** 1 Department of Occupational Therapy, University of Texas Medical Branch, Galveston, Texas; 2 Division of Rehabilitation Sciences, University of Texas Medical Branch, Galveston, Texas; 3 Department of Health Services, Policy, and Practice, Brown University, Providence, Rhode Island; 4 Division of Physical Therapy, Department of Rehabilitation Medicine, Emory University, Atlanta, Georgia; 5 Department of Preventive Medicine and Community Health, University of Texas Medical Branch, Galveston, Texas

## Abstract

**Introduction:**

Older Mexican Americans are living longer with multiple chronic conditions (MCCs). This has placed greater demands on caregivers to assist with basic activities of daily living (ADL) or instrumental activities of daily living (IADL). To understand the needs of older Mexican-American care recipients, we examined the impact of MCC on ADL and IADL limitations.

**Methods:**

We analyzed data from 485 Mexican American care-receiving/caregiving dyads. Selected MCCs in the analysis were diabetes, hypertension, stroke, heart disease, arthritis, emphysema/chronic obstructive pulmonary disease, cognitive impairment, depression, and cancer. Care recipients were dichotomized as having 3 or more conditions or as having 2 or fewer conditions. Three comorbidity clusters were established on the basis of the most prevalent health conditions among participants with comorbid arthritis and hypertension. These clusters included arthritis and hypertension plus: diabetes (cluster 1), cognitive impairment (cluster 2), and heart disease (cluster 3).

**Results:**

Care recipients with 3 or more chronic conditions (n = 314) had higher odds of having mobility limitations (OR = 1.98; 95% CI, 1.34–2.94), self-care limitations (OR = 2.53; 95% CI, 1.70–3.81), >3 ADL limitations (OR = 2.00; 95% CI, 1.28–3.17), and >3 IADL limitations (OR = 1.88; 95% CI, 1.26–2.81). All clusters had increased odds of ADL and severe ADL limitations. Of care recipients in cluster 2, those with arthritis, hypertension, and cognitive impairment had significantly higher odds of mobility limitations (OR = 2.33; 95% CI, 1.05–5.24) than those with just arthritis and hypertension.

**Conclusion:**

MCCs were associated with more ADL and IADL limitations among care recipients, especially for those with hypertension and arthritis plus diabetes, cognitive impairment, or heart disease. These findings can assist in developing programs to meet the needs of older Mexican-American care recipients.

## Introduction

Older adults residing in the United States are projected to become more racially and ethnically diverse in the next 40 years. The Hispanic population is the largest minority group, comprising 17.6% of the total US population (1). The Hispanic population aged 65 years or older numbers 3.1 million and is anticipated to reach 15.4 million by 2050 (2).

Mexican Americans are the largest Hispanic population in the United States (3). On average, older Mexican Americans are socioeconomically disadvantaged and are more likely than non-Hispanic whites to have chronic health conditions, including osteoarthritis, diabetes, hypertension, stroke, and cognitive decline (4,5). Despite these socioeconomic and health disadvantages, Hispanics have a longer life expectancy than non-Hispanic whites, which is called the Hispanic Paradox (6). This paradox is greatest among foreign-born Mexican Americans; because of their self-selection, individuals born in Mexico who migrate to the United States tend to have better health characteristics than those who stay in Mexico. This positive health selection may in part contribute to lower mortality rates for foreign-born Mexican Americans than their African American and white counterparts because of heart disease, smoking, and other causes (6,7).

*Familismo*, a cultural practice of many Hispanics, refers to emotional attachment and strong sense of loyalty and solidarity among family members (8). This practice contributes to older Hispanics being less likely than non-Hispanic whites to use formal health care services such as nursing homes, long-term care, or home health services (9–11). Hence, they place increased demands on their family members for routine and complex tasks.

Aging predisposes people to a high risk of developing multiple chronic conditions (MCCs) (12). Many studies identified combinations of multiple chronic conditions (13–17) and examined their impact on activities of daily living (ADL) and instrumental activities of daily living (IADL) limitations among older adults (18,19). Studying the relationship between multiple chronic conditions and the functional characteristics of older Mexican Americans is important given this population’s long life expectancy, high prevalence of chronic health conditions, and dependence on family care. An understanding of the impact of multiple chronic conditions on assistance needs among older Mexican Americans is crucial to plan appropriate health care delivery.

The objective of this analysis was to examine the impact of multiple chronic conditions on the ADL and IADL limitations of older Mexican Americans. We also explored potential differences in the ADL and IADL limitations of older Mexican Americans with different combinations of multiple chronic conditions. 

## Methods

### Sample population

We used data from the seventh observation wave of the Hispanic Established Populations for the Epidemiologic Study of the Elderly (HEPESE). Detailed descriptions of the sampling procedures and data collection techniques of the HEPESE are provided elsewhere (20). HEPESE is an ongoing longitudinal study of older Mexican American adults living in Texas, New Mexico, Colorado, Arizona, and California. The baseline observation wave (1993–1994) included 3,050 Mexican Americans aged 65 or older; 9 total observations were completed as of 2016. During wave 7 (2010–1011), 1,078 older adults were interviewed, 925 of whom provided contact information for an informant who was the primary person they would go to for advice or help with things they are unable to do by themselves. The informant reported characteristics of the respondent (eg, health, financial status, functional abilities) and provided information on assistance required by the respondent to complete ADLs and IADLs. The institutional review board at the University of Texas Medical Branch approved the HEPESE study before data were collected.

Because this study focused on care recipients who needed assistance from caregivers to complete self-care and household tasks, respondents who reported they were independent in self-care and household tasks (n = 329) were excluded from this study. Studies using data from the HEPESE used a similar approach to selecting study care recipients who needed assistance (21). Fifty-seven care recipients were also excluded from the sample because they did not receive the Mini Mental Status Exam, and 54 care recipients were excluded because of missing information for sociodemographic or health characteristics. Thus, 485 care-recipient/caregiver dyads comprised the final sample (**Figure**).

**Figure:**
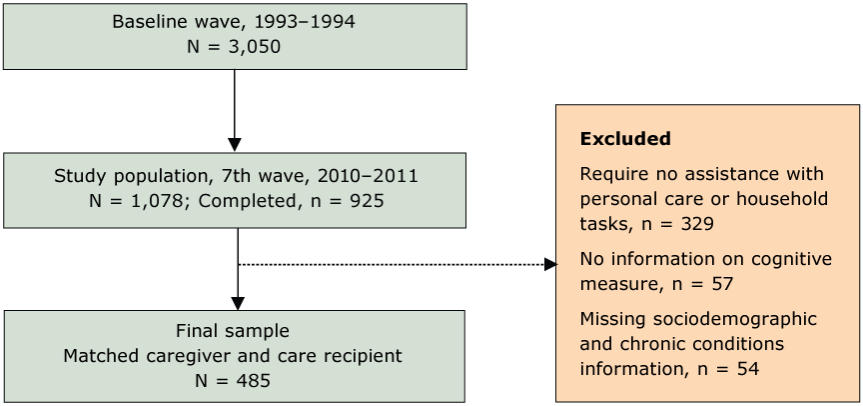
Flowchart showing exclusion criteria for study on effect of multiple chronic conditions on activity limitations among Mexican Americans, Hispanic Established Population for the Epidemiologic Study of the Elderly, 2010–2011.

### Measures

Sociodemographic measures collected from the care recipients were age, sex, years of education completed, and marital status (married, widowed, not married). Chronic conditions consisted of diabetes, hypertension, stroke, heart disease, arthritis, emphysema/chronic obstructive pulmonary disease, cognitive impairment, high depressive symptoms, and cancer. These health conditions were selected on the basis of previous literature (13–15,18,19) and health conditions included in the HEPESE questionnaire. These conditions, with the exception of depression and cognitive impairment, were identified based on self-report of the care recipient. Care recipients with a systolic blood pressure of 140 mm Hg or higher, or a diastolic blood pressure of 90 mm Hg or higher were also classified as having hypertension. Care recipients with high depressive symptoms were identified by a score of 16 points or higher on the Center for Epidemiologic Studies Depression (CESD) Scale (22). Cognitive impairment was defined as scoring 17 points or lower on the Mini Mental Status Exam (23).

Multiple chronic conditions is commonly defined as the presence of 2 or more health conditions (24). Preliminary analysis of the 485 care recipients included in the final sample indicated that 73 (15.1%) had fewer than 2 chronic conditions and only 15 (3.1%) had no chronic conditions. In the United States, adults with 3 or more chronic conditions make up only 28% of the total population but contribute to 67% of total health care expenditures (25). On the basis of findings from our preliminary analysis, we divided care recipients into 2 groups: those with 2 or fewer chronic conditions (reference group) or those with 3 or more chronic conditions.

We also identified 3 comorbidity clusters to investigate whether ADL and IADL limitations of care recipients varied according to the prevalence of individual combinations of chronic health conditions. The comorbidity clusters were created by using a multistep process. First, we identified the 2 most common health conditions among care recipients in the final sample. Consistent with research, arthritis and hypertension were the most common (18,26). Next, we identified care recipients who had comorbid arthritis and hypertension (n = 281). Finally, we identified the 3 most common health conditions among the 281 care recipients with comorbid arthritis and hypertension. This resulted in the three comorbidity clusters, which were arthritis and hypertension plus: diabetes (Cluster 1; n = 132), cognitive impairment (Cluster 2; n = 90), and heart disease (Cluster 3; n = 111). These comorbidity clusters were analyzed separately, because some care recipients could be included in more than one cluster. For example, a care recipient with arthritis, hypertension, diabetes, and heart disease would be included in Cluster 1 and Cluster 2.

Interviewers asked caregivers if the care recipient required assistance from a person (either the caregiver or another person), special equipment, or both, to complete ADL and IADL. ADL included walking across a small room, bathing, grooming, dressing, eating, getting from a bed to a chair, and using the toilet. IADLs included using a telephone, driving a vehicle or traveling alone, shopping, preparing a meal, doing light housework, taking medication, and managing finances. ADL items were grouped into mobility tasks (walking across a small room, getting from a bed to a chair) and self-care tasks (bathing, grooming, dressing, and eating). Severe ADL limitations and severe IADL limitations were defined as being unable to complete more than 3 ADLs and more than 3 IADLs, respectively.

### Statistical analysis

We used analysis of variance and χ^2^ statistical tests to compare the sociodemographic and health characteristics of care recipients with 3 or more health conditions to those with 2 or fewer health conditions. We conducted multivariable logistic regression models to estimate the odds of care recipients having limitations in one or more mobility tasks or self-care tasks, severe ADL limitations, and severe IADL limitations, according to the 3 comorbidity clusters. All analyses adjusted for the age, sex, education, and marital status of the care recipient. All statistical analyses were performed using R, version 3.1.0 (R Foundation) (27).

## Results

Overall, the mean age of the care recipients was 86.2 years, 66% were female, and the mean years of education completed was 4.5 years (**Table 1**). Arthritis and hypertension were both present in most care recipients. Diabetes, cognitive impairment, and heart disease were observed for approximately 30% of care recipients. Approximately 10% of care recipients reported having had a stroke or having been diagnosed with emphysema/chronic obstructive pulmonary disease (COPD) or cancer. Compared with care recipients with 2 or fewer health conditions, those with 3 or more health conditions were more likely to be female, completed fewer years of education, and were less likely to be married (**Table 1**).

**Table 1:** Demographic and Health Characteristics of Study Participants, Hispanic Established Population for the Epidemiologic Study of the Elderly, 2010–2011^a^

Characteristic	Total Sample (N = 485)	Number of Health Conditions		*P* Value
		≤2 (n = 171)	≥3 (n = 314)	
**Age, mean (SD), y**	86.2 (4.2)	85.9 (4.0)	86.3 (4.3)	.42
**Sex**				
Male	165 (34.0)	68 (39.8)	97 (30.9)	.04
Female	320 (66.0)	103 (60.2)	217 (69.1)	
**Education, mean (SD), y**	4.5 (3.7)	5.5 (4.0)	3.9 (3.5)	<.01
**Marital status**				
Married	141 (29.1)	59 (34.5)	82 (26.1)	0.13
Widowed	303 (62.5)	97 (56.7)	206 (65.6)	
Not married	41 (8.5)	15 (8.8)	26 (8.3)	
**Health condition**				
Diabetes	186 (38.4)	11 (6.4)	175 (55.7)	<.001
Hypertension^b^	394 (81.2)	105 (61.4)	289 (92.0)	<.001
Arthritis	332 (68.5)	70 (40.9)	262 (83.4)	<.001
Stroke	50 (10.3)	3 (1.8)	47 (15.0)	<.001
Heart disease	163 (33.6)	16 (9.4)	147 (46.8)	<.001
High depressive symptoms^c^	141 (29.1)	9 (5.3)	132 (42.0)	<.001
Cognitive impairment^d^	159 (32.8)	32 (18.7)	127 (40.4)	<.001
Emphysema/COPD	56 (11.5)	4 (2.3)	52 (16.6)	<.001
Cancer	45 (9.3)	4 (2.3)	41 (13.1)	<.001
**Number of limitations**				
>3 ADL	153 (31.5)	36 (21.1)	117 (37.3)	<.001
>3 IADL	280 (57.7)	78 (45.6)	202 (64.3)	<.001
**Number of health conditions**				
0	15 (3.1)	—	—	—
1	58 (12.0)	—	—	—
2	98 (20.2)	—	—	—
3	121 (24.9)	—	—	—
4	101 (20.8)	—	—	—
≥5	92 (19.0)	—	—	—

Abbreviations: — , not applicable; ADL, activities of daily living; COPD, chronic obstructive pulmonary disease; IADL, instrumental activities of daily living; SD, standard deviation.

a Values are expressed as no. (%) unless otherwise indicated. Care recipients could have more than one health condition.

b Hypertension defined as systolic blood pressure of ≥140 mm Hg or diastolic blood pressure of ≥90 mm Hg.

c High depressive symptoms were defined as scoring ≥16 points on the Center for Epidemiologic Studies Depression (CESD) Scale (22).

d Cognitive impairment was defined as scoring ≤17 points on the Mini Mental Status Exam (23).

Fifteen care recipients (3.4%) had zero health conditions and 92 (19.0%) had 5 or more health conditions (**Table 1**). Of all care recipients, 314 (64.7%) had 3 or more chronic conditions. Hypertension, arthritis, and diabetes (Cluster 1) were present in 132 care recipients; 90 had hypertension, arthritis, and cognitive impairment (Cluster 2); and 111 had hypertension, arthritis, and heart disease (Cluster 3). A total of 253 care recipients had limitations for mobility tasks, 255 had limitations for self-care tasks, 153 had severe ADL impairment, and 280 had severe IADL impairment.

Of total study participants (N = 485), 171 (35.3%) had 2 or fewer chronic health conditions, and 314 (64.7%) had 3 or more chronic health conditions (Table 1). The 2 groups did not differ significantly in age, but did differ in the remaining variables, including sex, education, marital status, and presence of the following health conditions: diabetes, hypertension, arthritis, stroke, heart disease, high depressive symptoms, cognitive impairment, emphysema/COPD, and cancer. Those in the 3 or more chronic health conditions were more likely to have limitations in ADL and IADL. Compared with care recipients with 2 or fewer health conditions, those with 3 or more were more likely to be female, to have completed fewer years of education, and were less likely to be married.

Results of the multivariable logistic regression indicated that care recipients with 3 or more chronic conditions had significantly higher odds compared with care recipients with 2 or fewer chronic conditions of having limitations in one or more mobility tasks (odds ratio [OR] = 1.98; 95% confidence interval [CI], 1.34–2.94), self-care tasks (OR = 2.53; 95% CI, 1.70–3.81), and to have severe ADL limitations (OR = 2.00; 95% CI, 1.28–3.17) and severe IADL limitations (OR = 1.88; 95% CI, 1.26–2.81) (**Table 2**). Among the 3 comorbidity clusters, cluster 2 (arthritis, hypertension, cognitive impairment) was the only cluster associated with significantly higher odds of limitations in one or more mobility tasks (OR = 2.33; 95% CI, 1.05–5.24). All 3 comorbidity clusters were associated with increased odds of limitations in self-care tasks, severe ADL limitations, and severe IADL limitations. Comorbidity cluster 3 (arthritis, hypertension, heart disease) was associated with the highest odds of limitations in self-care tasks (OR = 5.67; 95% CI, 2.57–13.00), whereas comorbidity cluster 2 (arthritis, hypertension, cognitive impairment) was associated with the highest odds of severe ADL limitations (OR = 4.51; 95% CI, 1.83–12.41) and severe IADL limitations (OR = 4.36; 95% CI, 1.91–10.25).

**Table 2:** Impact of Multiple Chronic Conditions on ADL and IADL Limitations, Hispanic Established Population for the Epidemiologic Study of the Elderly, 2010–2011^a^

Comorbidity Status	Mobility Tasks		Self-Care Tasks		>3 ADL Limitations		>3 IADL Limitations	
	OR (95% CI)	*P* Value	OR (95% CI)	*P* Value	OR (95% CI)	*P* Value	OR (95% CI)	*P* Value
**No. of health morbidities**								
≤2	1 [Reference]							
≥3	1.98 (1.34–2.94)	<.001	2.53 (1.70–3.81)	<.001	2.00 (1.28–3.17)	.002	1.88 (1.26–2.81)	.002
**Cluster 1 (n = 132)**								
Arthritis and hypertension	1 [Reference]							
Arthritis, hypertension, and diabetes	2.07 (0.99–4.38)	.05	3.72 (1.75–8.22)	<.001	3.98 (1.67–10.66)	.003	3.20 (1.50–7.03)	.003
**Cluster 2 (n = 90)**								
Arthritis and hypertension	1 [Reference]							
Arthritis, hypertension, and cognitive impairment	2.33 (1.05–5.24)	.04	4.23 (1.88–9.89)	<.001	4.51 (1.83–12.41)	.002	4.36 (1.91–10.25)	<.001
**Cluster 3 (n = 111)**								
Arthritis and hypertension	1 [Reference]							
Arthritis, hypertension, and heart disease	1.89 (0.89–4.02)	.10	5.67 (2.57-13.00)	<.001	3.98 (1.66–10.73)	.003	2.95 (1.36–6.58)	.007

Abbreviations: ADL, activities of daily living; CI, confidence interval; IADL, instrumental activities of daily living; OR, odds ratio.

a All models adjusted for demographic characteristics (age, sex, education, and marital status) of care recipients. Three comorbidity clusters were established on the basis of the most prevalent health conditions among participants with comorbid arthritis and hypertension. These clusters included arthritis and hypertension plus: diabetes (cluster 1), cognitive impairment (cluster 2), and heart disease (cluster 3). Care recipients could belong in more than one cluster.

Results from the comparative analysis of the 3 comorbidity clusters indicated that only cluster 2 (arthritis, hypertension, cognitive impairment) had a significantly higher chance of needing assistance with mobility tasks. Multiple chronic conditions were associated with more ADL and IADL limitations, especially for care recipients with hypertension and arthritis plus diabetes, cognitive impairment, or heart disease (**Table 2**).

## Discussion

We found that care recipients with 3 or more chronic conditions needed caregiving assistance with mobility, self-care, and ADL- and IADL-related tasks, regardless of their combination of chronic conditions. Studies show that older Mexican Americans often have multiple chronic conditions (28) that can limit their ability to live independently (12). Given a choice, many aging Americans would prefer to stay in their homes and communities rather than be placed in nursing homes, a concept known as “aging in place.” The older Mexican American population is no different in their preferences; however, they tend to rely on family members for their health care needs rather than using local formal health care resources. The use of health care, including hospital stays, physician visits, and costs, increases among adults with multiple chronic conditions (26,29).

Our study showed that all 3 chronic condition clusters were associated with increased odds of limitations in self-care tasks, severe ADL limitations, and severe IADL limitations. Care recipients with arthritis, hypertension and heart disease (cluster 3) were most likely to require assistance with basic self-care tasks. A study reported that unpaid caregivers caring for individuals with heart disease perceived they themselves had difficulty in performing tasks such as household cleaning and management of bills and finances (30). Older adults with cognitive impairment have higher risk of mobility impairment and physical decline (31,32), which can further complicate their care. Furthermore, older adults with cognitive impairment often require supervision to prevent injury even if they are able to perform some self-care or household tasks.

Because older adults are living longer, their informal caregivers will most likely age and experience health problems and functional impairments themselves, which may contribute to greater burden for caregivers. Moreover, caring for older Mexican Americans with multiple chronic conditions, especially those with cognitive impairments, can be challenging. Informal caregivers serving a minority population tend to provide more and higher intensity of care than caregivers serving nonminority populations (33). A previous study determined that caregivers of Mexican Americans who had more involved mobility impairments, limitations in IADLs, depressive symptoms, and cognitive decline were more likely to have psychological distress (21). Similarly, another study found that caregivers of Mexican Americans aged 70 years or older had fewer physician visits than their non-caregiving counterparts (34). Among new caregivers of older Mexican Americans, those who had high levels of acculturation (ie, who have lived in the United States for a longer duration) were more likely to report depressive symptoms at 2 years after the start of caregiving than those with lower levels of acculturation (35).

Our study has limitations. Our sample was restricted to 5 southwestern US states and may not be representative of other growing populations of older Hispanics in other US locations. Our inclusion criteria for this analysis resulted in the most impaired older Mexican Americans being selected, which may have inflated the percentage of the final sample with hypertension, diabetes, and heart disease. This analysis focused on older Mexican Americans who had limitations in daily activities, and we excluded those who did not have limitations in ADLs or IADLs. This exclusion may have influenced our results since older Mexican Americans with multiple chronic conditions, but who did not need assistance in daily activities, were excluded from the final sample. The chronic conditions identified in the HEPESE data set were based on self-report from care recipients and could be subject to recall bias. Furthermore, many chronic health conditions that may affect ADL and IADL functioning (eg, atrial fibrillation, high cholesterol) were not collected in HEPESE. Finally, participants who did not provide contact information for an informant could not be included in our analysis, which may have influenced our results; these participants may not need help from another person and thus may be in better health than participants who did provide information for an informant. Alternatively, participants who did not provide contact information for an informant may have less social support than participants who did provide contact information for an informant.

Our analysis also has strengths. First, the HEPESE is a well-characterized cohort of older Mexican American adults that includes measures for a range of sociodemographic, health, and functional characteristics. Second, data for ADL and IADL limitations of the care recipient were collected from an informant (informal caregiver) who has insight of the functional characteristics and health care conditions of the care recipient.

Many older Mexican-American care recipients are living with multiple chronic conditions. Our analysis provides evidence that older Mexican American care recipients with 3 or more health conditions are highly likely to have severe ADL and IADL limitations and difficulty completing self-care and mobility tasks. Care recipients with comorbid arthritis and hypertension along with diabetes, cognitive impairment, or heart disease may be most likely to have functional limitations. Our findings can ensure that resources are appropriately allocated to caregivers on the basis of physical and cognitive impairments of older Mexican-American care recipients.
